# Diaries of Nursing Students during the COVID-19 Pandemic: A Qualitative Descriptive Study

**DOI:** 10.3390/ijerph18168556

**Published:** 2021-08-13

**Authors:** Serpil Türkleş, Münevver Boğahan, Hilal Altundal, Zeliha Yaman, Mualla Yılmaz

**Affiliations:** Department of Mental Health Nursing, Faculty of Nursing, Mersin University, 33343 Mersin, Turkey; munevverbghn@mersin.edu.tr (M.B.); hilalaltundal@mersin.edu.tr (H.A.); zyaman15@mersin.edu.tr (Z.Y.); mualley69@mersin.edu.tr (M.Y.)

**Keywords:** COVID-19, diary, nursing, students, experiences, feelings, qualitative study

## Abstract

Little is known about the experiences of nursing students during the pandemic process. This research was conducted to determine the feelings, thoughts, and experiences of nursing students during the COVID-19 pandemic process. This qualitative study was conducted with 47 first-year nursing students of a faculty that experienced the COVID-19 pandemic between 3–30 April 2020. Student nurses stated that they felt fear and anxiety; they liked this situation in the beginning due to the constraints during the pandemic process, but due to the prolongation of this process, they experienced boredom due to monotonous extraordinary days of doing the same things every day and realized that every moment before the pandemic was very valuable. In addition, the students stated that rich and poor are equal in the face of the virus and that all humanity has learned solidarity by leaving wars, fights, and superiority efforts. In this process, it was found that nursing students have negative coping methods, such as not being able to manage time well due to constraints at home and spending too much time on the phone, internet, and computer. In this context, empowering nursing students to cope with challenging emotions and thoughts starting from their educational life will contribute to the development of both students and the profession.

## 1. Introduction

The new coronavirus disease (COVID-19) appeared in Wuhan in December 2019 and spread all over the world in a short time [1,2,3]. A pandemic was declared by the World Health Organization (WHO) on 11 March 2020 [4]. In our country, as in other countries, various protective measures have been taken in social life, business, and education such as travel restrictions, social distance, and isolation [3,5]. Within the scope of protective measures, Turkey’s Higher Education Council (YOK) suspended education for three weeks in all higher education institutions as of 16 March 2020, and distance education period was started [6].

The spread of the disease, isolation measures, and the closure of all schools and universities in the country as well as the suspension of education negatively affect the academic performance and mental health of students [3,7]. Students may experience difficulties such as fear of becoming infected, taking care of their friends and families who are sick, changing the structure of education and assessment methods, and difficulties in adaptation to the change in regular teaching methods [8,9]. In parallel with this, there has been an increasing interest in the mental health of university students all over the world [10,11,12].

In the COVID-19 pandemic period, nursing students have been among the groups that experienced the most difficulties due to their practical lessons being held in hospitals [13]. When the literature is examined, there are studies on distance education of nursing students during the COVID-19 pandemic [14,15,16], their mental health [17], perceived stress, views on COVID-19 [18], anxiety levels, perspectives on the nursing profession [19], emotional responses and coping strategies [20,21], and preventive, traditional, and complementary medicine practices against COVID-19 [22]. There are a limited number of qualitative studies examining the experiences of student nurses during the pandemic process and their feelings and thoughts about the pandemic process. In one of these qualitative studies, the tweets of student nurses on social media were examined. It was stated that students tweeted about reactions to the COVID-19 pandemic, daily life, student role, social connections, and sociopolitical issues [23]. In other studies, how student nurses perceive COVID-19, their experiences in this process [24], the difficulties they encounter during distance education [25,26], and the experiences of senior nursing students who joined the health care workforce during the pandemic process were examined [27]. In our study, unlike other studies, first-year nursing students who have not yet experienced professional practice were asked to keep a diary between specific dates during the pandemic process, and their experiences in this process were examined. It is thought that the interruption of education, the transition to the distance education, the uncertainty experienced, the interruption of clinical practices, and their first encounter with the pandemic have negatively affected the students who are still in the process of adapting to the profession and the university. It is thought that revealing the experiences of nursing students, who will have important roles in healthcare practices in the future, during the pandemic, will make a significant contribution to handling the possible effects of the pandemic on the students, the management of the difficulties that may occur after the pandemic, and the appropriate interventions. This study was conducted to identify the experiences of first-year nursing students studying at the Faculty of Nursing of a university in Turkey during the first month of social restrictions of the COVID-19 pandemic.

## 2. Materials and Methods

### 2.1. Type of Research

This study is qualitative research. The Consolidated Criteria for Reporting Qualitative Research Checklist (COREQ) was used in the reporting of the study [28].

### 2.2. Population and Sample of the Study

The population of the research consists of first-year students at the Faculty of Nursing of a university that experienced the COVID-19 pandemic between 3–30 April 2020 (*N* = 195). Between 3–30 April 2020 is the first month of social restrictions in our country. In particular, we wondered what the students who had not yet taken vocational courses were experiencing during the first month of social restrictions of the COVID-19 pandemic. For this reason, first-year students were determined as the population of the study, as they had not yet taken practical courses specific to the profession and had no hospital experience. The sample group consisted of 47 students who volunteered to participate in the study on the specified dates. The sample group consisted of 47 students because 6 students or their relatives became sick, 9 students filled their diaries incompletely, and 5 students stated that they did not want to continue. To determine the sample size for qualitative studies, a sampling approach is used that requires researchers to continue collecting data until sizes adequate for the saturation point are reached [29]. The sample group consisted of 47 students, and data saturation was achieved. The encodings of the participants are given in Table 1.

### 2.3. Collection of Data

In order to examine the experiences of first-year nursing students, students were asked to keep a diary between 3–30 April 2020 and to include their feelings, thoughts, and experiences regarding the coronavirus pandemic and send them to the researchers via e-mail. In qualitative studies that include diaries, explanations about how the participants fill the diaries are included. An announcement has been made to students about the guidelines for writing a diary. In the announcement, the students were requested:

- To keep a diary in a 1-week period.
- To explain how they spent their daily lives during the social isolation process.
- To write their diary at the end of the day.
- To explain their feelings and thoughts in a plain language during the COVID-19 pandemic process.
- To transfer what they wrote to the computer and send it via e-mail.
- Not to write down their identity information (for ease of expressing their true feelings and thoughts).

A preliminary practice was applied to five students to evaluate the availability of the data collection form (pilot study). Because no arrangement was made after the preliminary practice of the data collection form, the five students were also included in the study. Field notes record were not taken. No repeat diaries were required. Transcripts were not returned to participants for comment and/or correction. The interviews were conducted in Turkish and then translated into English.

### 2.4. Ethical Aspect of the Research

Ethical approval for this study was obtained from the Social Sciences Ethics Committee of the university (2020/35). Institutional permission from the relevant Faculty was obtained. In addition, written and verbal consent was obtained from the nursing students during data collection. The current regulations on data protection or online security have been complied with. It explained to students that despite sending the email, these data would not be rescued. As stated in the Law on the Protection of Personal Data in Turkey, it is stated that data should be protected in transactions such as recording, storing, preserving, changing, rearranging, disclosing, transferring, taking over, making available, classifying, or preventing. However, it is also stated in the law that the processing of personal data for purposes such as research, planning, and statistics by anonymization with official statistics can be carried out with the informed written consent of the persons [29].

### 2.5. Analysis of Data

Data analysis has been carried out according to eight stages of content analysis developed by Downe-Wamboldt (1992) at the same time as data collection. The content analysis encompasses the following steps: (1) selecting the unit of analysis, (2) creating and defining the categories, (3) retesting the category definitions, (4) assessing reliability and validity, (5) revising the coding rules, (6) retesting the revised category scheme, (7) coding all the data, and (8) reassessing reliability and validity. We employed inductive content analysis to generate important meanings regarding the experience elicited from the individuals’ perspective. No software program was used for the qualitative data analysis. In the qualitative analysis of the data transferred to the digital environment, a content analysis was carried out by taking into account the prevalence of the comments contained in the responses beyond the words, the number of participants who made the same interpretation and used the same word, what was meant to be said, and the originality of the answers. The diaries written by the students were combined considering the differences and similarities. After the raw data generated were carefully read by each researcher, the data were processed (coding meaningful concepts and themes). Themes were created by gathering the encoded data. During the analysis of the obtained data, raw data were given to two field experts who have researched on qualitative studies, and an expert opinion was obtained [28,30].

### 2.6. Research Team, Reflexivity, and Rigor

The researchers have been working as research assistants (3 PhD students) and faculty members (Professor and Associate Professor) in a nursing faculty in the Department of Mental Health and Psychiatric Nursing. They have worked as nurses/supervisors in hospitals in the past. The researchers consisted of five female researchers who were trained in qualitative research and worked experience. Five of the researchers are working as academicians at the university where the research was conducted. Therefore, they have prior relationships with the students in the sample group.

At the beginning of the diaries, the researchers stated that the participant would be able to explain her views freely and that every view was valuable. The same e-mail address and form were used in all diaries to maintain consistency. All diaries were collected by one researcher. Diaries were terminated at the point of satisfaction. To provide credibility, the opinions of the participants are presented with explanatory notes in the results section.

## 3. Results

The average age of the students participating in the study is 19.80 ± 0.77 (lower-upper values = 18–21); 85.1% (*n* = 40) of the students are women.

Table 2 shows the themes obtained from the diaries of nursing students during the first month of social restrictions of the COVID-19 pandemic.

### 3.1. Theme 1. Nursing Students’ Emotions during the COVID-19 Pandemic

#### 3.1.1. Fear and Anxiety

This is example 1 of an equation: It was found that these two emotions were the most intense emotions that all of them experienced. It was stated that they watch the news of death in the country and the world every day, and they experience a lot of worry, anxiety, and fear. They stated that they were horrified by only watching the news about COVID-19 in the news bulletin, emphasizing that “the circle is narrowing” and that the fear increased even more when the news of the death of their relatives began to come. They stated that the reason for the fear and anxiety was having little information about the virus, the fear of being infected and their relatives being infected, and the uncertainty of what will happen in the future. They stated that the rapid spread of the virus in the world and in our country caused panic, and they were sad due to not being able to see their friends and relatives due to having to stay at home. It was emphasized that the process of removing clothes, bathing, washing hands every minute, and constant cleaning as soon as arriving home started in all segments of the society due to the fear and anxiety, and the number of people taking public transport has decreased. It was reported that during the process that started with lockdown and restrictions, there was anxiety and fear due to loss of freedom. The statements of students regarding this sub-theme are given below.

“*Of course, I was most afraid when my father was going to work. Every day until my father came home, I was always thinking about him out of fear that he would be infected too.”*

(P38, F, 21)

“*This disease mostly affected the elderly and patients with weak body resistance. I was worried about my mother. Because she is both older, has low body resistance, and a heart disease. I hope this disease will end without anything happening to my mother. I’m getting very scared.”*

(P33, F, 21)

#### 3.1.2. Boredom

Students stated that they liked being at home at the beginning due to the restrictions during the pandemic, but they started to feel bored from doing the same things every day as the process became longer. They described how the daily routines started to become the same and the boredom they experienced due to a monotonous life, not enjoying life, starting to lose the meaning of life, and the unprecedented days everyone is going through. In addition, there were students who stated that their psychological health had deteriorated and they were experiencing despair and unhappiness.

“*This disease made people weak and irritable. The longer you get stuck at home, the more you feel overwhelmed.*”

(P33, F, 21)

“*Every day became the same. There is nothing we can do. We are stuck at home*.”

(P9, F, 20)

#### 3.1.3. Longing for the Past and Hope for Beautiful Days in the Future

Students stated that they realized that they were living freely in the pre-pandemic period and that they remembered their past memories and good days by talking about them with their friends. They stated that they longed for times spent in cafes and restaurants before the pandemic, face-to-face classes, and hospital practices at school, days when they walked freely on the street and did sports and went to concerts and movies. They also emphasized that they realized during the pandemic that every moment they had before the pandemic was very valuable. Students expressed that they dreamed of returning back to the good old days as soon as possible. The statements of students regarding this sub-theme are given below.

“*It seems that the breaths we took, going outside, going out with friends, breathing in the air were great freedoms. When we return to our normal lives, these will be much more valuable for all of us. Who would say that we would miss the school, the desks*.”

(P8, F, 20)

“*Wishing to write diaries willed with hope again in beautiful days when there is less virus and no new cases.*”

(P12, F, 18)

### 3.2. Theme 2. Nursing Students’ Views on Pandemic

#### 3.2.1. The Meaning COVID-19 Added to Life and What It Taught Humanity

Students stated that they learned the meaning of life during the pandemic, that individuals who do not follow the restrictions and rush to the markets behave irresponsibly; they are happy when they hear the recovered and discharged patients, and when they see that hugging, a very important element in our culture, is over, they understood how valuable it is to touch and hug someone. They stated that they witnessed people suffering from economic difficulties, losing their jobs, and being unable to attend funerals. It has been stated that rich and poor are equal in the face of the virus, and that all humanity has learned solidarity by leaving wars, fights, and superiority efforts. In this context, they emphasized that the whole world should fight together against the pandemic, and the nature polluted by humans was recovering during the pandemic. The statements of students regarding this sub-theme are given below.

“*Today I learned very clearly that hugging is an organ like my hand, and how it is actually a beautiful freedom to smell the sea scent that we pollute all the time. It turns out that hugging is a powerful and beautiful thing. The biggest damage this disease caused is not being able to hug.*”

(P12, F, 18)

“*Air pollution has also decreased, the waters have cleared, and the depletion of the ozone layer has slowed down. If this virus ends, I hope one day it will end, a clean world awaits us for us to pollute.*”

(P40, F, 19)

#### 3.2.2. Nursing Students’ Views on Nurses Working during the Pandemic

Students stated that during the pandemic, healthcare workers could not go home for months and worked devotedly, healthcare professionals were applauded in the society, gained value, and the nursing profession became more visible. They stated that they want to become nurses as soon as possible and help healthcare professionals in this process and that they are proud of their profession. The statements of students regarding this sub-theme are given below.

“*While watching the news, I learned that Cemil Taşcıoğlu (he is the first doctor to die in Turkey due to COVID-19) passed away and I was very sad as a healthcare practitioner candidate. We owe a lot to all healthcare professionals who work through their lives, once again I am proud of the profession I have chosen and am studying*.”

(P42, M, 20)

“*As a nursing student, I wish I could do something. I wish I could help sick people right now, like millions of healthcare professionals. I want to stand by the patients in their struggle and witness their victories*.”

(P37, F, 20)

### 3.3. Theme 3. Nursing Students’ Coping Strategies during the Pandemic

#### 3.3.1. Effective Coping Strategies of the Nursing Students

It was found that more than two-thirds of students used positive coping strategies during the pandemic. Some students stated that they used effective coping methods such as looking at old photo albums with family members, telling memories and laughing, sending old photos to friends through WhatsApp and making conversations through video calls, spending time with family members (playing backgammon, rummikub), praying, reading books, gardening, and relaxing.

“*We see this time as an opportunity for creative activities and conversations on different topics instead of being sad at home. Thus, we spend quality and fun time. So that we can stay away from worrying constantly as a family.*”

(P47, F, 18)

“*It will be ensured that they understand better what kind of a thing the virus is in fun ways by pictures, drawing shapes, and watching animations. We cannot be very effective if we get angry with little kids who are unaware of this situation for touching some places or simply by telling them. Today, I think I raised awareness of my nephews without boring them with the animations I show them. We also had a fun time.*”

(P43, F, 20)

#### 3.3.2. Ineffective Coping Strategies of the Nursing Students

It was found that almost one-third of students used negative coping strategies during the pandemic. They stated that they could not manage their time at home well due to restrictions, spent too much time with technology such as cell phones, internet, and computers, gained weight due to continuously sleeping, cooking, and eating, and they could not cope well during this process.

“*Yesterday, four hours permission was given for children under 20 to go out. But I couldn’t go out because I fell asleep that day, and this really upset me.*”

(P46, F, 20)

“*I washed my hands and face, I had my breakfast. When the thought of not being able to leave the house came to my mind, it was as if I aged 1 year. I wandered around the house a little later, sat in front of the computer, and continued my knee from the section I left off. I continued the series until noon, after eating lunch, I did not get up from the computer until the evening, as you know, there is not much to do.*”)

(P39, M, 19)

## 4. Discussion

It was found that during the pandemic, students experienced feelings of fear and anxiety most intensely and experienced fear, dread, anxiety, and panic regarding the news they watched about the pandemic every day. The reasons for fear and anxiety were found to be not having enough information about the virus, fear of being infected and their relatives being infected, and due to uncertainty about the future. Qualitative studies have described that nursing students experience stress and anxiety due to the uncertainty caused by the pandemic [26,30]. Studies conducted similarly revealed that university students experienced anxiety and depression during the COVID-19 pandemic [11,19,31]. Gao et al. (2020) emphasized in a study that there is a positive relationship between the prevalence of anxiety and depression and following COVID-19 news on social media [32]. In addition, in the studies conducted, there are reasons that cause anxiety and stress in university students such as fear for their own and their loved ones’ health, fear of getting COVID-19, general uncertainty about the pandemic, uncertainty about the future of their career, delays in academic activities, and relatives or acquaintances being infected with COVID-19 [11,33].

The students stated the whole society took some measures due to fear and anxiety in order to decrease the spread of the disease such as taking their clothes off as soon as arriving home and taking a shower. Literature findings indicate that nursing students experience fear [24,25] and follow the rules by showing seriousness and professionalism in preventing the spread of the virus [24,25,34]. Studies have shown that students have basic information about COVID-19 transmission and common symptoms, they changed their behavior in accordance with public health recommendations (increase in hand washing frequency, wearing a mask), and stopped doing some daily practices (for example, leaving home, shaking hands, social contact, and meetings) [18,35,36].

Students said that due to the restrictions, their freedom has disappeared, and they experienced anxiety and fear; they could not see their friends and relatives due to having to stay at home and they felt sadness. Patelarou et al. (2021) state in their study on the COVID-19 epidemic that quarantine and social isolation had an effect on the mental health of nursing students, and priority should be given to providing university-based mental health interventions [17]. In a study conducted with university students who were quarantined at home, the prevalence of post-traumatic stress disorder was 2.7% and depression was 9.0%. In addition, feeling extreme fear, reduced sleep time, and living in severely affected areas have been shown to be important risk factors for psychological distress [37]. Majrashi et al. (2021), in their systematic review, found that the COVID-19 period was stressful and worrying for nursing students and affects their mental health [38]. The literature is in parallel with the results of this study. Efforts to mitigate the detrimental effects of COVID-19 on students’ psychological health should be prioritized.

It was found that students stayed at home in accordance with the restrictions, but as the process became longer, their daily routines started to become the same, and they experienced boredom due to a monotonous life, not enjoying life, and beginning to lose the meaning of life due to the unprecedented days everyone is going through. In addition, some students stated that their mental health was negatively affected and they experienced despair and unhappiness. Aristovnik et al. (2020) emphasize that university students faced problems with online learning, they felt inadequate, and they experienced boredom, anxiety, and disappointment about their future professional careers and studies [39]. In a study, it was reported that 26.63% of the participants had clinically significant psychological distress and 11.10% met the criteria for a possible acute stress reaction [10]. In a study, it was reported that nursing students experienced moderate level of stress, but their stress scores were higher compared to the previous period, students stayed at home during the day due to the curfew imposed on people under 20 years of age, and the majority (66.2%) experienced boredom at home [18]. Okuyan et al. (2020) emphasized that nursing students have high levels of health anxiety during the pandemic, they are adversely affected by staying at home due to the pandemic, they feel overwhelmed and nervous, they are afraid of infection and death and feel anxious and overwhelmed about the future [40]. The results of our research are compatible with the literature.

The students stated that they realized that they lived freely in the pre-pandemic period, that they longed for moments spent in cafes and restaurants, face-to-face classes, and hospital practices at school, days when they walked freely on the street and did sports and went to concerts and movies. They stated that they made video calls with their friends, talked about their memories, and dreamed about returning back to the good old days with hope. Wang et al. (2020) reported that university students experienced anxiety regarding the changes in social relationships and social isolation, increased social/physical distance, and decreased social activities due to isolation [2]. Okuyan et al. (2020) emphasized in a study they conducted that the situations that affected the nursing students the most during the COVID-19 pandemic are, respectively, withdrawal from entertainment and social life, decreased contact with family, relatives, colleagues, and neighbors, and being away from school [40]. The results of our study are similar to those in the literature.

The students stated that they learned the meaning of life during the pandemic and understood how valuable it is to touch and hug someone. They stated that they witnessed people suffering from economic difficulties, losing their jobs, and being unable to attend funerals. It is thought that psychological strains such as uncertainty, death news, restriction of freedom, fear, and anxiety that are experienced during the pandemic may cause the meaning of life to be questioned and lead people to search for meaning. Sami et al. (2020) state that there are positive changes in the majority of young people, such as the meaning of life, a more meaningful and positive view of life, and paying more attention to their relationships with people compared to the past. In addition, it is emphasized that they became aware of many values such as living, spiritual resources, social activities, freedom, health, family, the importance of patience, friends, and hugs [41]. Lovric et al. (2020) states that nursing students accept the importance of human togetherness [24]. These results support the results of our study.

The students stated that healthcare workers worked devotedly during the pandemic, were applauded in the society, and gained value. They wanted to become nurses as soon as possible and help healthcare professionals in this process, and they were proud of their profession. It is thought that the importance of the nursing profession has increased during the pandemic, that it became more visible in the society and the media, and the support of the public for healthcare professionals has positively affected the perceptions of nursing candidates regarding the profession. In the studies conducted, nursing students stated that their experiences during the pandemic improved them, they want to start working to provide healthcare services, and the support given to nurses by the public positively reinforces their career choices [42,43,44]. Taş et al. (2020) reported that 79.1% of nursing students stated that the COVID-19 pandemic affected their motivation, and 52.5% stated that their motivation was negatively affected. In addition, it is emphasized that the students’ motivation is negatively affected due to lack of practical courses, distance education, the prestige of the nursing profession has increased, and their motivation has increased in relation to the realization of the importance of the nursing profession [45]. Cici et al. (2021) reported a decrease in the number of nursing students with a positive view of the nursing profession, and an increase in those with negative and undecided views about the profession. It was emphasized that the anxiety scores of those who have a negative view of the profession and those who do not want to have this profession in the future have significantly increased during the pandemic [19]. Velarde-García et al. (2021), in their qualitative study, found that nursing students have difficulty in applying their knowledge and skills because they lack a defined role and competence [27]. In a study conducted in Turkey, they stated that nursing students could not acquire professional skills because they could not practice the vocational courses, and their skills regressed, causing a lack of self-confidence and a sense of inadequacy [26]. Qualitative studies have reported that nursing students feel responsible for their own society, want to take responsibility in nursing roles that support a holistic approach with their knowledge and skills, and once again, understand the importance, responsibilities, and risks of the nursing profession [24,25]. It has been observed that the results of the study differ in the literature. It was found that the students used positive and negative coping methods during the pandemic. This result suggests that students who have negative coping behaviors are at risk in terms of their physical and mental health. In the literature, they reported that nursing students had difficulty in accepting the situation at first and were unprepared, but then they adapted. Students reported that they coped with using different psychological and situational coping strategies, using social media networks in their relationships with colleagues and peer groups, planning their basic daily routines as a stress management tool, healthy eating, physical exercise or planning rest periods, entertainment, humor, and religion [46]. In addition, it is stated that students use the strategies of seeking information and counseling, staying optimistic, and coping with transference [38]. Wang et al. (2020) state that university students experienced problems such as changing eating and sleeping habits, insufficient exercise, and weight gain [2]. In a study, it was stated that 15.84% of university students indicated that they could not cope with stress and benefit from health services. Students experienced problems such as a change in eating and sleeping habits, insufficient exercise, weight gain, excessive alcohol intake, isolation, and crying [47]. Son et al. (2020) found that 23% of university students used negative coping methods such as ignoring the news of COVID-19, sleeping longer, distracting them by doing other things, alcohol, and smoking [33]. In a study, 42.8% of the students had healthier eating behavior during the pandemic, and 2.9%, 6.3%, and 69.7% of the students, respectively, used “unchanged or more” smoking, drinking, and physical activity [12]. When the literature is reviewed, it is reported that nursing students had moderate coping behaviors during the COVID-19 pandemic [12,48]. In a study, it was stated that approximately half of the participants were able to cope with stress sufficiently; 15.84% could not cope or use healthcare services [2]. When the studies are examined, it is seen that university students used positive coping methods such as communicating with their family and friends, healthier eating behavior, meditation and breathing exercises, spiritual measures, maintaining routines, positive reframing, physical exercise, using social media, playing with pets, keeping a diary, listening to music, praying, reading, gardening, and drawing [2,33,49,50].

### Strengths and Limitations

Since this research is qualitative, the results cannot be generalized for all nursing students. The research is limited to nursing students studying at a university and the questions in the form. The size of the study group and the different characteristics of the participants constitute the strengths of the study. While collecting the data, the voluntary basis was emphasized in participation in the study. Correct and complete answers were obtained by establishing a relationship based on mutual trust with the participants. In this study, both the coding and the determination of the themes were carried out and compared separately by three researchers. A common vision has been reached as to which piece of data belongs to which code. After all data were coded, the coding was sent to two field experts of qualitative research, and an expert opinion was obtained. All researchers contributed to the analysis and writing process.

## 5. Conclusions

It was found that nursing students experienced feelings and thoughts such as fear, anxiety, despair, unhappiness, boredom, not enjoying life, stress during the pandemic, were negatively affected by staying at home, and felt overwhelmed and tense. It was found that nursing students used positive and negative coping methods during the pandemic. Nursing students who had difficulties in coping are at risk in terms of physical and mental health. In this context, empowering nursing students to cope with challenging emotions and thoughts starting from their educational life will contribute to the development of both students and the profession. It is thought that these findings obtained from the diaries of nursing students during the COVID-19 pandemic will guide them and the nursing profession in the following years. These diaries will guide nursing students in both their private and professional lives when there are other pandemics. Nurse educators can give nursing students educations about self-knowledge, self-awareness, anxiety and coping with difficult situations, healthy use of social media, making use of free time correctly and effectively, and recognizing and managing emotions.

## Figures and Tables

**Table 1:** Students’ characteristics (*n* = 47).

Participants No	Gender	Age	Participants No	Gender	Age
P1	Male	20 years	P25	Female	20 years
P2	Female	20 years	P26	Male	20 years
P3	Female	20 years	P27	Female	21 years
P4	Female	20 years	P28	Female	20 years
P5	Female	20 years	P29	Female	20 years
P6	Female	20 years	P30	Female	19 years
P7	Female	20 years	P31	Female	21 years
P8	Female	20 years	P32	Female	19 years
P9	Female	20 years	P33	Female	21 years
P10	Male	19 years	P34	Female	20 years
P11	Female	19 years	P35	Female	20 years
P12	Female	18 years	P36	Female	20 years
P13	Female	20 years	P37	Female	20 years
P14	Female	21 years	P38	Female	21 years
P15	Female	19 years	P39	Male	19 years
P16	Female	21 years	P40	Female	19 years
P17	Female	20 years	P41	Female	20 years
P18	Female	20 years	P42	Male	20 years
P19	Male	20 years	P43	Female	20 years
P20	Male	20 years	P44	Female	19 years
P21	Female	18 years	P45	Female	21 years
P22	Female	20 years	P46	Female	20 years
P23	Female	19 years	P47	Female	18 years
P24	Female	19 years			

**Table 2:** Themes obtained from the diaries of nursing students during the first month of social restrictions of the COVID-19 pandemic.

Theme	Sub-Theme
Theme 1. Nursing students’ emotions during the COVID-19 pandemic	Fear and anxiety. Boredom. Longing for the past and hope for beautiful days in the future.
Theme 2. Nursing students’ views on pandemic	The meaning COVID-19 added to life and what it taught humanity. Nursing students’ views on nurses working during the pandemic.
Theme 3. Nursing students’ coping strategies during the pandemic	Effective coping strategies of the nursing students. Ineffective coping strategies of the nursing students.

## Data Availability

Written and verbal consent was obtained from the nursing students during data collection.
